# Optimising Evidence-Based Psychological Treatment for the Mental Health Needs of Children with Epilepsy: Principles and Methods

**DOI:** 10.1007/s10567-019-00310-3

**Published:** 2020-01-21

**Authors:** Roz Shafran, Sophie Bennett, Anna Coughtrey, Alice Welch, Fahreen Walji, J. Helen Cross, Isobel Heyman, Alice Sibelli, Jessica Smith, Jamie Ross, Emma Dalrymple, Sophia Varadkar, MICE Research Study Team, Rona Moss-Morris

**Affiliations:** 1 Population, Policy and Practice Research and Teaching Department, University College London Great Ormond Street Institute of Child Health, 30 Guilford Street, London, WC1N 1EH UK; 2 Great Ormond Street Hospital NHS Foundation Trust, Great Ormond Street, London, WC1N 3JH UK; 3 Health Psychology, Institute of Psychiatry, Psychology and Neuroscience, Kings College London, London, SE1 9RT UK; 4 King’s College London, Strand, London, WC2R 2LS UK; 5 Department of Primary Care and Population Health, UCL Medical School (Royal Free Campus), University College London, Rowland Hill Street, London, NW3 2PF UK

**Keywords:** Epilepsy, Anxiety, Depression, Behaviour, Implementation science

## Abstract

There are potent evidence-based psychological treatments for youth with mental health needs, yet they are rarely implemented in clinical practice, especially for youth with mental health disorders in the context of chronic physical illness such as epilepsy. Implementation science, the study of the translation of research into practice, can promote the uptake of existing effective interventions in routine clinical practice and aid the sustainable integration of psychological treatments with routine health care. The aim of this report was to use four implementation science methods to develop a version of an existing effective psychological treatment for mental health disorders [the Modular Approach to Treatment of Children with Anxiety, Depression or Conduct Problems (MATCH-ADTC)] for use within paediatric epilepsy services: (a) literature search; (b) iterative focus groups underpinned by normalisation process theory; (c) Plan–Do–Study–Act methods; and (d) qualitative patient interviews. Findings: Three modifications were deemed necessary to facilitate implementation in children with both mental health disorders and epilepsy. These were (a) a universal brief psychoeducational component addressing the relationship between epilepsy and mental health; (b) supplementary, conditionally activated interventions addressing stigma, parental mental health and the transition to adulthood; and (c) additional training and supervision. The intervention needed relatively little alteration for implementation in paediatric epilepsy services. The modified treatment reflected the scientific literature and the views of clinicians and service users. The multi-method approach used in this report can serve as a model for implementation of evidence-based psychological treatments for children with mental health needs in the context of other chronic illnesses.

## Introduction

Epilepsy is one of the most common serious long-term illnesses in young people, with a lifetime prevalence of 1% (Russ et al. 2012). Children with epilepsy are significantly more likely than those without physical health disorders to experience a multitude of severe and chronic mental health disorders including depression, anxiety, attention-deficit hyperactivity disorder and disruptive behaviour disorders (Davies et al. 2003) which affect quality of life (Baca et al. 2011) and potentially impact the epilepsy itself (Hesdorffer et al. 2012). Such mental health difficulties also impair educational attainment, are costly and have enduring effects into adulthood (Fastenau et al. 2008; Murphy and Fonagy 2012). Despite decades of international research and policy guidelines that emphasise the importance of identifying and addressing the mental health needs of youth with epilepsy (Plioplys et al. 2007), and the availability of evidence-based psychological treatments for mental ill-health, their mental health needs continue to be undetected and undertreated (Asato et al. 2014; Ott et al. 2003).

There are a number of potential explanations for the failure of implementation of effective psychological treatments for mental health problems in children with epilepsy. These explanations can be seen at the individual clinician, service and organisational levels. For example, clinicians who are skilled in the treatment of epilepsy are typically medically trained neurologists and clinical nurse specialists; the interventions for epilepsy are predominantly anticonvulsant medications, surgical interventions and device implantations (National Institute for Health and Care Excellence [NICE] 2012). The clinicians delivering psychological interventions are typically psychologists or counsellors with little training in the medical aspects of care. Therefore, many individual clinicians do not have the requisite knowledge and skills to treat both epilepsy and mental health problems. At the service level, services are contracted (and funded) to treat either the neurological condition or the mental health condition. Organisations addressing mental health are often entirely separate from those treating physical health and epilepsy, despite their widely accepted close relationship (World Health Organisation [WHO] 2018). Despite these obstacles, there are many advantages to attempting to integrate psychological treatment into the package of care offered by paediatric epilepsy services. These include improved identification of mental health needs, better access to evidence-based psychological treatment, provision of patient-centred care, avoiding fragmentation of health services, reducing stigma associated with mental health treatment, optimising both mental health and physical health outcomes and strengthening overall health systems (Patel et al. 2013). Integration is needed as psychological interventions targeting the enhancement of health-related quality of life, medication adherence and comorbid mental health symptoms (for example anxiety, depression and disruptive behaviour) have been recommended as part of comprehensive epilepsy care (Michaelis et al. 2018).

Integration of services could be obtained by having a mental health specialist embedded in the team, as recommended for the treatment of diabetes (Young-Hyman et al. 2016) and illustrated by the national ‘Improving Access to Psychological Therapies—long-term conditions’ programme in the UK (McCrae et al. 2015). Although attractive in principle, accessing psychological therapies is often challenging and there are insufficient therapists to meet current demands (Mental Health Taskforce 2016), often resulting in long waiting lists (Smith et al. 2018). An alternative approach is to train existing clinical nurse specialists or neurologists working in epilepsy services with a special interest in mental health to deliver evidence-based psychological interventions. This alternative approach has the advantage of the clinician having expertise in both the physical and mental health aspects of epilepsy and truly being able to deliver a patient-centred approach to optimise health outcomes. However, clinicians are typically extremely busy and may not wish to have additional responsibilities beyond their core practice.

Regardless of whether a mental health specialist is embedded in the epilepsy team or whether existing epilepsy clinicians are trained to deliver psychological interventions, the question arises as to the optimal psychological intervention for children with epilepsy and mental health needs. There are established, evidence-based treatments for the most common mental health disorders that arise in conjunction with epilepsy including depression (Klein et al. 2007), anxiety (Higa-McMillan et al. 2016) and disruptive behaviour disorders (Leijten et al. 2018). Although clinical trials of the effectiveness of these treatments in children who also have epilepsy are sparse, the evidence that does exist suggests similar effectiveness of interventions for children with chronic illness as those without (Bennett et al. 2015; Corrigan et al. 2016; Law et al. 2019; Moore et al., 2019) and therefore existing interventions should be the starting point for addressing mental health needs in young people with epilepsy.

One psychological therapy that shows promise is the Modular Approach to Treatment of Children with Anxiety, Depression or Conduct Problems (MATCH-ADTC) which was created to combine procedures from the evidence-based treatment manuals for depression, anxiety, behavioural difficulties and trauma into one system that can address multiple presenting difficulties (Chorpita and Weisz 2009). The intervention has been shown to be effective in two trials conducted by the developers (Chorpita et al. 2017; Weisz et al. 2012) with the rate of improvement for youths in the modular approach being significantly better than for those in usual care at 2-year follow-up (Chorpita et al. 2013), although there is some other recent indication that it may not be superior to usual care (Weisz et al. 2019). Of critical importance for sustained implementation, there is an excellent, empirically supported supervisory model to accompany the intervention (Weisz et al. 2018) and factors affecting its implementation in routine community settings have been identified (Cheron et al. 2019). Additionally, practitioners were satisfied by the intervention (Chorpita et al. 2015) and continued to use it in routine practice after training and consultation ended (Palinkas et al. 2013; Thomassin et al. 2019). Other psychological treatments for specific disorders, e.g. the Incredible Years Parent Training (Menting et al. 2013) or transdiagnostic approaches (Marchette and Weisz 2017) incorporating evidence-based interventions, could also be candidates for use in children with epilepsy and mental health disorders. However, the ability to address multiple presenting problems is essential for young people with epilepsy who tend to have multiple mental health comorbidities (Reilly et al. 2014). The modularity offered by MATCH-ADTC, accompanied by excellent training and patient materials, led us to conduct a preliminary evaluation of MATCH-ADTC in young people with epilepsy (Bennett et al. 2018). This preliminary work indicated that it is feasible to deliver MATCH-ADTC to this population in a self-help format via the telephone (Bennett et al. 2018) and highlighted the importance of the intervention being delivered flexibly within epilepsy services using a patient-centred approach. However, the preliminary work led to important questions about how best to modify and optimise the intervention for children with epilepsy for delivery within epilepsy services by non-mental health specialists.

## Aims and Objectives

The current research aimed to optimise MATCH-ADTC for use in children and young people with mental health needs in the context of epilepsy within routine epilepsy services, using implementation science methods. The objective of this report is to describe the principles of using these methods to modify the intervention with the view that such principles are generalisable to the use of any evidence-based psychological therapy in children with a comorbid long-term condition.

## Methods and Results

**Fig. 1:**
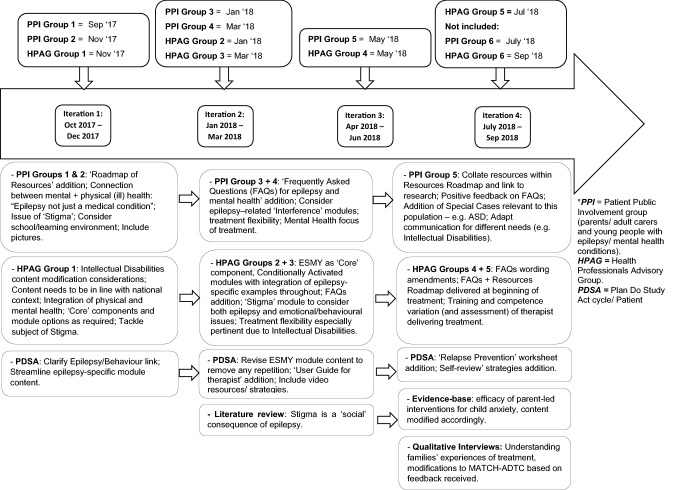
Summary of the development of the intervention across time using the four methods

Four related implementation science methods were used to develop a version of MATCH-ADTC delivered via the telephone for use in children and young people with epilepsy that could be implemented within routine epilepsy services. The methods were undertaken concurrently and the process of intervention was iterative by design, with the information yielded by each individual method feeding into the execution and results of the others over the course of one year. This process is documented in Fig. 1.

### Literature Review

An informal review was conducted to identify key relevant psychological treatment strategies considered important to address the mental health of children with epilepsy. The Medline, PsychInfo and Embase databases were searched with the following terms (exploded): Epilepsy + Mental Health (Anx, Dep, Conduct disorders) + Child (/adolescent) + Illness Perceptions. ‘Illness perceptions’ was included as a search term based on the importance of the ‘common sense model of illness’ (Leventhal et al. 1980) which helps understand reactions and responses to illness. The focus was on quantitative research and was confined to the English Language. The target result was to identify key psychological interventions that may need to be incorporated into the MATCH-ADTC intervention. Searches were conducted for literature published between 2007 and 2017.

Prominent researchers in the fields of epilepsy and child mental health were contacted to ensure that all recent relevant research was identified. The literature searches and contact with researchers identified five key areas that were identified as important for incorporation into a specific version of MATCH-ADTC for young people with epilepsy. The first area was psychoeducation. Psychoeducation is incorporated into a range of effective psychological interventions in both physical and mental health (e.g. Barsevick et al. 2002; Tursi et al. 2013) and the original MATCH-ADTC provides education about the nature of anxiety, depression and behavioural problems. Additional psychoeducation about the close relationship between epilepsy and mental health (e.g. Davies et al. 2003) was therefore considered a necessary addition to the intervention. The second area highlighted in the research literature was the importance of both epilepsy-related and mental health-related stigma and its associated impact on well-being and mental health (e.g. Jacoby and Austin 2007; Kaushik et al. 2016). Third, the literature and contact with researchers highlighted the need to incorporate an option of parent-delivered therapy for the anxiety module with younger or developmentally delayed children (e.g. Smith et al. 2014; Thirlwall et al. 2013); the behavioural module within MATCH-ADTC is largely parent-led already. However, the literature also highlighted that parents of children with epilepsy were at high risk of suffering from anxiety and depression (Reilly et al. 2018) and therefore the fourth area of importance was to address parents’ own mental health needs within the modified approach. Finally, the literature review and contact with researchers identified the particular challenges facing adolescents with epilepsy with transition to adulthood (and adult services) including the consumption of alcohol, sleep deprivation and feeling different from peers, for example being unable to drive (Camfield et al. 2012).

### Focus Groups

Patient–public involvement (PPI) and co-production are essential for implementation to ensure the research and clinical strategies meet the needs of the population served and are key to consideration of patient preferences which is a fundamental aspect of evidence-based practice (Bombard et al. 2018; Sackett et al. 1996). Therefore, six iterative focus groups of children and young people were held. These were chaired by ED (parent and study lead for PPI) and comprised 10 self-selected parents/carers and 5 young people (age 6–14 years; 3 males) with epilepsy and experience of mental health services who had received treatment for epilepsy. Details of the contributors are described in Table 1, with representation across developmental age, epilepsy type and mental health needs.

**Table 1:** Summary of Participant characteristics across the three methods

Child demographics	PPI Group (*n* = 9 families; 5 young people; 10 parents; 2 grandparents)	Qualitative Interviews (*n* = 7 interviews with *n* = 8 parents)	Plan–Do–Study–Act Cycles
Sex	2 Female, 7 male	5 Female, 2 male	7 Female, 5 male
Age range	6–14	5–16	5–18
Epilepsy types	Range of seizure types including focal and generalised Range of seizure frequencies from monthly to multiple daily Dravet syndrome (*n* = 1)	Range of seizure types including focal and generalised Range—seizure free over a year to multiple daily seizures Genetic related (*n* = 1)	Range of seizure types including focal and generalised Range—seizure free over a year to multiple daily seizures Genetic epilepsies (*n* = 3)
Special educational needs?	8	7	11
Additional diagnoses recorded in clinical record?	Physical: Vision problems (*n* = 1), kidney dysfunction (*n* = 1) Specific learning difficulties: Dyslexia (*n* = 2) Neurodevelopmental: ADHD (*n* = 3), ASD (*n* = 3)	Physical: Cerebral Palsy (*n* = 2), autoimmune condition (*n* = 1), hemiplegia (*n* = 2) Neurodevelopmental: ASD (*n* = 2), ADHD (*n* = 1)	Physical: Hemiplegia (*n* = 2) Cerebral Palsy (*n* = 2) Leukodystrophy (*n* = 1) Neurodevelopmental: ASD (*n* = 3), ADHD (*n* = 1)

*ADHD* Attention-Deficit Hyperactivity Disorder, *ASD* Autism Spectrum Disorder

Topics covered included issues of engagement, questionnaire completion and delivery of the intervention across the age range. Parent and child groups were held separately. Normalisation Process Theory (NPT) was used as a framework to guide discussion topics about the mental health needs of children with epilepsy, modifications to the MATCH-ADTC intervention and barriers to implementation. NPT proposes that ‘complex interventions become routinely embedded (implemented and integrated) in their organisational and professional contexts as the result of people working, individually and collectively, to implement them’ (Murray et al. 2010). It describes four constructs that lead to interventions or work practices becoming “normalised”, that is becoming embedded into routine practice: coherence (‘sense making’), cognitive participation (building relationships with stakeholders), collective action (the operational work of putting the new intervention into practice) and reflexive monitoring (appraising the new practice), each of which have four further sub-constructs (Murray et al. 2010). For example, we asked questions about how the intervention is different from previous treatments received, their understanding of the purpose and value of the MATCH-ADTC intervention, and their views about how it could be implemented within epilepsy services.

The children and young people and parent focus groups made a number of important, specific suggestions to improve the intervention. An illustration of these can be seen in Table 2. Of note, children, young people and parents considered that from the start of the intervention, it was essential for therapists to help families separate the child from the disorder and to provide links to additional resources. There was a consensus that while many families wish to receive information about the multiple difficulties associated with epilepsy including, but not limited to, mental health problems, other families may find too much information overwhelming so links to reputable resources would enable individual families to see additional information in their own time at their own pace. There were extended discussions about the stress of parenting a young person with mental health needs in the context of epilepsy and it was considered essential for there to be a method by which such parental stress could be addressed within the treatment when it was a barrier to progressing with their treatment (e.g. the parent was too depressed to be able to implement the behavioural strategies). There was also consensus about the need to provide additional support to some families whose children were growing up and transitioning to adult services and adulthood.

**Table 2:** Amendments to the intervention based on suggestions from the Research Advisory Group

PPI RAG suggestion	Amendment based on suggestion
Did not like the original ‘MESY’ acronym	Changed the name of the additional epilepsy-specific module to ‘ESMY’ (‘Epilepsy-Specific Module for Youth)
Epilepsy-specific content within all of the modules will ensure it is relevant and families will feel it is tailored to them	Included epilepsy-specific examples and modifications throughout the manual
Epilepsy treatment is “already in place” and therefore they would prefer if the therapy could start as soon as possible without weeks of epilepsy information at the beginning	Have one session at the start (ESMY) which explores the link between epilepsy and mental health and then begin the mental health treatment
After diagnosis—did not know what resources were available, felt overwhelmed with information, many had to teach themselves and collate their own information from a variety of sources	Created a ‘Frequently Asked Questions’ handout Several amendments were made to this handout based on further discussion and suggestions by the group Created a ‘Roadmap of Resources’ handout. Several amendments were made to this handout based on further discussion and suggestions by the group
Helpful to have the therapist consider the positive aspects of the child early on in treatment	Direct quotes from this discussion, i.e. “Epilepsy is not just a medical condition” and “You are not your epilepsy” were incorporated into the therapist script for the assessment session
Important to include information on autism and ADHD	Added autism and ADHD to the ‘special cases’ sections in the manual Included information in the ‘Roadmap of Resources’
Stress is the most important issue to address for parental mental health	Created a parental mental health module and included a progressive muscle relaxation within this
Helpful to have information presented in more than one way	Videos were added to supplement the handouts and worksheets
Children with epilepsy have many comorbidities so it is important the therapist is able to accommodate for these	Added a section in the therapist user guide which explicitly states that the intervention needs to be tailored to the child and family and provides suggestions on how to do this
YP group indicated that anger was a dominant emotion they felt	Included consideration of anger in the ‘Learning to Relax’ module
Strong feelings regarding the therapist’s use of terminology (i.e. seizures vs fits) and how they are addressed by the therapist (i.e. mum vs. Ms. Smith)	Created a form for families to complete at the start of therapy giving their preferences regarding terminology, how to be addressed and additional comments

Six iterative focus groups of health professionals working in epilepsy services were also held. These were chaired by SV, a consultant paediatric neurologist, and comprised 12 epilepsy/mental health/child specialists working in health and education (five males). Professionals were selected to represent psychiatry, neurology, psychology, paediatrics, clinical nurse specialists, primary care, intellectual disability specialists, transition support workers and special educational needs teaching staff. The topics were the same as the PPI groups and the health professionals focus group provided input into the specific recommendations made by the children, young people and parent focus groups. Additionally, they considered the obstacles to implementation of the traditional MATCH-ADTC protocol (or any modified version) and the appropriateness of MATCH-ADTC considering the developmental and chronological ages of the patients typically seen in epilepsy services that go up to the age of 18 years. They highlighted the importance of transition to adulthood (and, relatedly, adult services) as a time of critical importance and viewed it as essential that the intervention should be able to take such transition into account.

Like the PPI focus groups, the health professionals group was also guided by NPT. The Normalisation Process Theory Toolkit (May et al. 2011) was used. This has statements defining each NPT variable (e.g. key individuals drive the intervention forward ‘whether or not key individuals are able and willing to get others involved in the new practice’) and a sliding bar for each statement allowing them to be rated according to the extent to which the statement is true or not true for the intervention/implementation in question (not at all—completely). A summary report with radar charts is produced which allows the user to identify areas of strength and potential weakness with the long-term sustainability of the intervention. The only area of weakness concerned organisational support for the perceived additional time that it would take services to address mental health needs alongside physical health ones as the mental health treatment sessions are not built into the job plans of the epilepsy nurses. In addition to the content of the intervention, the group considered that the traditional psychology supervision model, which is typically one hour a week, individually or in groups, was incompatible with the way that epilepsy nurse specialists work. It was agreed that a better model would be ‘supervision on demand’ whereby expert supervision from the research or clinical team was available whenever the therapist ran into difficulties, with a minimum of one scheduled supervision session per month.

### Plan–Do–Study–Act (PDSA) Cycles

Plan–Do–Study–Act (PDSA) methods (Berwick 1998) were used to make iterative tests of change to the MATCH-ADTC materials with twelve patients receiving the version of the MATCH-ADTC intervention. This is a key quality improvement method that is highly individualised and aims to maximise the feasibility and acceptability of interventions through providing a framework for developing, testing and implementing change (Taylor et al. 2014). Twelve patients (age 5–18 years; 8 white British; 5 males) with epilepsy and mental health needs were referred for treatment by paediatric epilepsy services at a national specialist hospital. They received a version of the MATCH-ADTC intervention and provided goal-based outcome (GBO) data as part of the usual clinical service. The full chronological and developmental age ranges were represented, as were the type of epilepsy and mental health needs. Epilepsy types included seizures with focal onset and generalised seizures; genetic epilepsies were included. Many of the children had additional diagnoses, such as cerebral palsy and leukodystrophy. Two had undergone neurosurgery. Six patients had been diagnosed with learning problems, ranging from specific learning difficulties to severe intellectual disability. Two had been noted to have characteristics of Autism Spectrum Disorder prior to the intervention. Presenting difficulties included behavioural difficulties, anxiety and depression.

Each iteration of the intervention involved a PDSA cycle which comprised ‘Plan’—planning the intervention or changed that will be implemented, ‘do’—conducting the change/intervention, ‘study’—investigating the extent to which the intervention/change worked and ‘act’—acting on the findings to make changes to the intervention until the results are in line with the original aims. In order to complete the ‘study’ phase of each cycle, weekly GBO data routinely used in mental health research and practice were collected for each participant to assess whether or not the intervention had been effective (Law and Jacob 2013). Each family set up to three goals for therapy and rated how much they thought they have moved towards these goals on a scale of 0–10, where 10 is the goal has been completely met and 0 is no progress has been made.

**Fig. 2:**
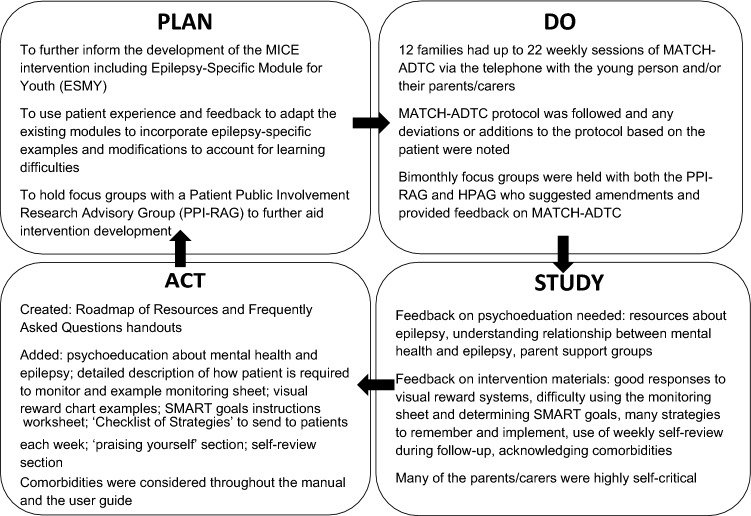
Summary of Plan–Do–Study–Act Cycles for the 12 patients who started the intervention

The PDSA cycles produced for each of the 12 patients who started the intervention are summarised in Fig. 2 These were informed by considerations of the effectiveness of the intervention through visual inspection of weekly scores on the GBO measures for nine participants. The mean goal rating at session 1 was 1.11 (SD = 0.31) and at the final session, it was 6.58 (SD = 1.78), where 10 = goal fully met. Of the remaining three, one completed an assessment only, one completed an assessment and one intervention session, and one completed six sessions but did not complete measures.

### Qualitative Interviews Methods and Results

Eleven families (13 parents) of the twelve who received the intervention were approached via email, telephone or letter to invite them to take part in a qualitative interview; one family was not approached due to known changes in family circumstances. Three families did not respond and one family declined participation due to lack of time. Of the five who did not take part in an interview, four had not completed the intervention (two required an interpreter and a further two had recently undergone neurosurgery). Seven families expressed interest and were given the opportunity to ask questions before deciding whether to take part. Informed consent was taken either in person or over the phone, depending where the interview took place. Overall, eight parents from these seven families agreed to take part in the study (seven mothers, one father). The interviews were developed to understand the experiences of the patients in depth and included open-ended questions and non-directive prompts to encourage parents to bring up issues that they felt were relevant to them. The interview guide was piloted with three members of the research team and the PPI focus group to ensure the clarity of the questions and prompts. Parents were free to pause or stop the interview at any time and they were given the option to receive the transcript of the conversation (only 2 families requested this). Two researchers conducted all interviews. Neither of the interviewers delivered the MATCH-ADTC treatment to the participants taking part in the current study. All transcripts were analysed using inductive thematic analysis following the guidelines developed by Braun and Clarke (2006).

**Fig. 3:**
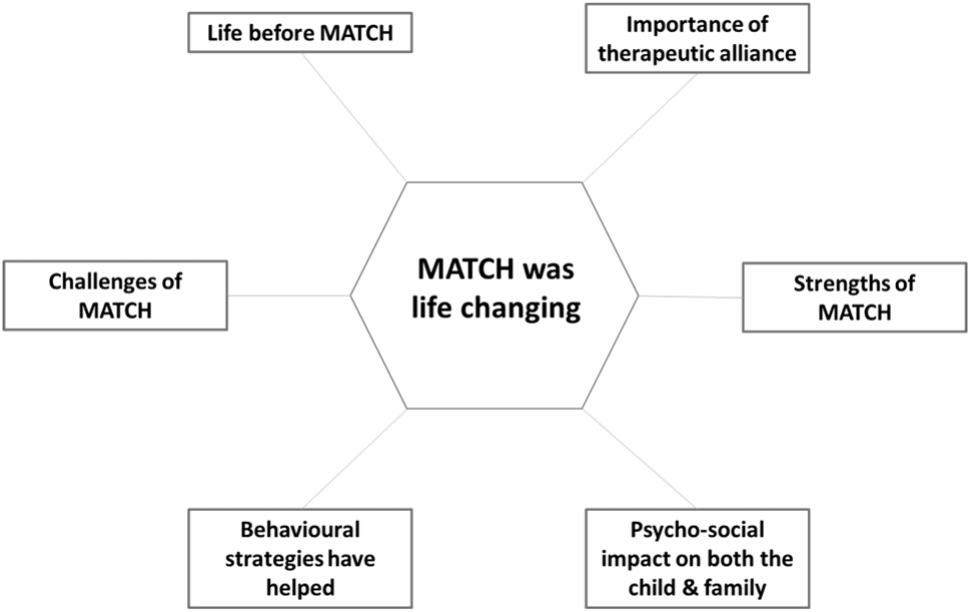
Main themes identified in qualitative interviews of 7 families who completed the intervention

Parents shared an array of views in relation to the treatment received. Firstly, they described receiving evidence-based modular psychological therapy in the form of MATCH-ADTC as life changing. This concept was a central theme linked to six further themes capturing their perceptions of the acceptability and relevance of MATCH-ADTC: life before MATCH-ADTC (focused on the lack of effective treatment options and support before receiving MATCH-ADTC), strengths of MATCH-ADTC (aspects of the treatment parents praised), challenges of MATCH-ADTC (features of the treatment that were perceived as difficult or challenging), behavioural strategies which helped (key processes through which MATCH-ADTC decreased the child’s difficulties), importance of the therapeutic alliance and psychosocial impact on the child and family (see Fig. 3). These themes mirrored those found in our earlier qualitative work using a version of MATCH-ADTC in this patient group (Bennett et al. 2018). Overall, parents in all iterations of the intervention described MATCH-ADTC as a treatment that changed the quality of life of both the child and the family. The treatment seemed appropriate despite initial iterations having no content adapted for children with epilepsy. Considering changes that needed to be made to the intervention, one parent in the first iteration acknowledged some barriers associated with telephone delivery, but they still praised the flexibility offered by phone sessions. Future iterations therefore included the option for face-to-face and/or Skype appointments. Though MATCH-ADTC was considered extremely beneficial, parents also discussed the aspects of the treatment that they found challenging, particularly the intensive nature of the intervention. Interestingly, these factors were recognised as inherent features of such a comprehensive treatment, rather than problematic situations that had to be changed to improve MATCH-ADTC. We therefore ensured that the assessment included clear and honest advice to families about the amount of time and effort required to undertake the intervention.

### Additional Factors to Consider

The literature review, focus groups, PDSA cycles and interviews indicated a need to personalise the intervention for the individual and context by (i) anglicising the language, (ii) using epilepsy-specific examples where possible (e.g. when discussing anxiety), (iii) making it explicit that the pace of the intervention and delivery may need to be adjusted on a case-by-case basis depending on the child’s intellectual ability and mode of delivery (telephone vs. face-to-face) and (iv) allowing the anxiety intervention to be delivered via parents when appropriate and preferred.

In order for the mental health intervention to be delivered within neurological services, the focus groups raised the issue of the need for additional training and supervision for staff. A five-day training course accompanied by a minimum of monthly supervision was therefore developed to ensure fidelity to the MATCH-ADTC model as well as facilitating delivery within busy services. A ‘user guide’ was also developed that makes it explicit how to use the manual for nurses and other health professionals unfamiliar with the delivery of mental health interventions. The training course was video-taped and made available online with all the materials to facilitate continued learning and training of new staff.

### Relationship Between the Methods

In order to optimise the learning arising from the methods, careful consideration was given to the timing of the literature review, focus groups, PDSA cycles and qualitative interviews. The literature review was conducted at the outset of the study. Weekly team meetings ensured that issues raised by the PDSA cycles and qualitative interviews were subsequently incorporated into the agendas for the focus groups and the emerging priorities from the focus groups subsequently influenced the PDSA cycles. The qualitative interview schedules were not explicitly changed by the findings from the focus groups or PDSA cycles. Combining NPT with PDSA cycles, a literature review, focus groups and qualitative interviews allowed the identification of potential barriers to implementation from the outset and was illuminating as there was a high level of consensus and substantial overlap. This multi-method approach to implementation ensured the voices of the patients, their families and the professionals across the disciplines were heard which is necessary for successful implementation. Use of several methods also allowed us to consider important aspects of implementation that may not have been considered had we only used one. For example, four of the five patients who did not participate in the qualitative interviews did not complete the treatment. However, we were able to reflect on their experiences of treatment through the use of PDSA cycles; two of these young people had recently undergone neurosurgery and two required interpreters.

### Implications for the Implementation of Psychological Therapies in Youth with Long-Term Conditions

Implementation science methods aim to bridge the gap between research and clinical practice. The issue of implementation needs to be considered from the outset of designing a psychological therapy and some interventions are easier to implement than others depending on factors such as treatment complexity, duration and training requirements. Modular interventions such as MATCH-ADTC have considerable advantages over non-modular interventions for the treatment of mental health disorders, including potential improvements in efficiency and effectiveness (Chorpita et al. 2005). One of the arguments for a modular design is that it should facilitate incremental improvements and ‘provides an *explicit framework for adaptation* without automatically dictating the adaptation of an existing protocol’ (Chorpita et al. 2005, p. 153). The current study provides an example of such adaptation. It was not necessary to design an entirely new treatment for young people with epilepsy but instead a protocol with empirical support was adapted within a year with additional modules developed to address the specific needs of young people with epilepsy. Modularity can facilitate the development of adaptations of psychological interventions for people with other chronic conditions, for example young people with cancer and low mood where it is clear that ‘one size doesn’t fit all’ (Coughtrey et al. 2019) and who might need a specific module to address issues such as learning to understand and describe low mood in the context of cancer (Reed-Berendt et al. 2019).

## Summary of Key Points

Together, the four methods identified that the following were required to optimise the use of MATCH-ADTC in children and young people with mental health needs in the context of epilepsy.

1. A core module for everyone that provides education about mental health disorders and their relationship with epilepsy, enables a formulation of the maintenance of mental health disorders within epilepsy, separates the child from the disorder and provides links to additional resources.
2. Additional ‘interference’ modules in keeping with the structure of MATCH-ADTC. These interference modules would be utilised when progress with psychological treatment was being impeded either at the service or patient level. The modules were as follows:
3. Stigma: Techniques to address stigma associated with mental health and epilepsy-related stigma (e.g. Heijnders and Van Der Meij 2006)
4. Parental Mental Health: Focus groups, PDSA cycles, interviews and the literature highlighted that parenting a child with epilepsy and mental health difficulties can be stressful (Reilly et al. 2018) and parental anxiety and depression were recognised as potential barriers for some families that needed to be addressed for successful implementation of the intervention.
5. Transition to adulthood: Such transition-related issues were considered as potential barriers to the implementation of the mental health intervention in this population and therefore necessary to address within the modified intervention when they arose.

## Conclusions

In conclusion, the multiple methods indicated that the interventions within the standard psychological treatment were largely suitable for use in a paediatric epilepsy service once the language had been anglicised and epilepsy relevant examples incorporated when possible, consistent with good clinical practice and flexible and personalised use of manuals (Hamilton et al. 2008). A clear model for training and supervision to ensure successful implementation by non-mental health specialists also emerged. This model, in conjunction with ensuring appropriate practical organisational support including time and technology resources, is likely to be important for overcoming some of the service and organisational barriers in implementation of evidence-based mental health interventions within physical health services in general. Our modified intervention will now be evaluated in a large, randomised controlled trial within epilepsy services (Trial Register number ISRCTN57823197).

The methods used in this study—literature review, focus groups with patients and practitioners, PDSA cycles and qualitative interviews—were complementary and generalisable and could be used to facilitate the implementation any psychological intervention (particularly modular ones), for young people with mental and physical health needs. The findings indicate that clinicians can be reassured that standard protocols can be applied and modified, if necessary, with appropriate input from colleagues, patients and the broader literature. Such an approach will hopefully facilitate increased use of these standard protocols for children with chronic illness whose mental health needs are currently being neglected.
